# A 40-Year Regional Study of Cleft Lip and/or Palate Incidence in Tokai, Japan

**DOI:** 10.7759/cureus.95191

**Published:** 2025-10-22

**Authors:** Teruyuki Niimi, Chisato Sakuma, Nagana Natsume, Hideto Imura, Kumiko Fujiwara, Ken Kitagawa, Hiroo Furukawa, Nagato Natsume

**Affiliations:** 1 Cleft Lip and Palate Center, Aichi Gakuin University Dental Hospital, Nagoya, JPN; 2 Oral and Maxillofacial Surgery, Osaka Medical and Pharmaceutical University Hospital, Osaka, JPN

**Keywords:** cleft lip and palate, congenital anomalies, epidemiology, japan, longitudinal study

## Abstract

Objective: Cleft lip and/or palate (CL/P) is among the most common congenital craniofacial anomalies worldwide, with reported incidences ranging from approximately 1 in 500 to 2,500 live births, depending on the geographic region and ethnic background. While genetic and environmental factors contribute to its etiology, long-term regional surveillance is essential to detect trends and identify risk factors. This study investigates the epidemiological characteristics of CL/P in the Tokai region of Japan over a 43-year period (1981-2023).

Methods: Since 1981, the Cleft Lip and Palate Center at Aichi Gakuin University Dental Hospital has been conducting an annual population-based monitoring survey involving all obstetric facilities in Aichi, Gifu, and Mie prefectures. Mailed questionnaires captured data on total live births, presence and types of congenital anomalies, CL/P occurrence and classification, sex, and any associated anomalies. Trends in annual incidence, cleft type distributions, sex ratios, and comorbid anomaly prevalence were analyzed.

Results: Over the 43-year period, the incidence of CL/P remained stable, ranging from 10 to 20 cases per 10,000 live births per year, with no statistically significant year-by-year variations. Cleft lip (CL) and cleft lip with palate (CLP) were more frequent in males, whereas isolated cleft palate (CP) was historically more common in females. After 2004, CP showed a reversal of the sex ratio, with more male cases than female (p < 0.001). Despite rising maternal age and social changes, no significant trend toward increasing CL/P incidence was observed. Similarly, the prevalence of other major congenital anomalies (e.g., cardiac, limb, chromosomal disorders), as reported through the annual regional survey beginning in 2009, remained stable through 2023.

Conclusion: This long-term regional surveillance study demonstrates a stable incidence of CL/P in the Tokai region in spite of demographic shifts, such as declining birth rates and rising maternal age. These findings emphasize the value of sustained, regionally based congenital anomaly monitoring systems in guiding health policy, perinatal counseling, and genetic risk assessment.

## Introduction

Cleft lip and/or palate (CL/P) ranks among the most frequently encountered congenital craniofacial malformations worldwide, with an estimated incidence of 1 per 500 to 2,500 live births [1-3]. Beyond its morphological implications, CL/P can cause significant functional challenges, including feeding difficulty, speech impairment, otitis media-associated hearing loss, dental and occlusal abnormalities, and psychological or social development issues, warranting early and sustained multidisciplinary care [4].

The incidence of CL/P is influenced not only by genetic predisposition but also by environmental exposures, ethnicity, geographic region, temporal trends, and the diagnostic/reporting capabilities of healthcare systems [5-8]. In addition, advances in prenatal diagnostic technology and the option of pregnancy termination can alter the apparent incidence reported at birth, although the extent of this influence varies across countries and cultural contexts. Consequently, detailed regional epidemiological surveillance is critical for evaluating incidence trends, identifying potential risk factors, and informing preventive strategies and health policy.

In Japan, regional variability in CL/P incidence has been observed. Social phenomena such as delayed marriage and advanced maternal age have been suggested as potential risk modifiers; however, to our knowledge, no studies have directly examined the association between maternal age and CL/P incidence in the Japanese population. In response, robust regional monitoring infrastructure and accurate dissemination of localized data have become academic and public health priorities.

Beginning in 1981, the Cleft Lip and Palate Center at Aichi Gakuin University Dental Hospital has led a regional epidemiological monitoring initiative encompassing all obstetric facilities in the three prefectures of Tokai (Aichi, Gifu, and Mie). This program, arguably one of Japan’s most enduring and comprehensive CL/P surveillance systems, has accrued over four decades of high-quality data, receiving significant recognition both nationally and internationally.

This manuscript presents an analysis of CL/P incidence in Tokai from 1981 to 2023. We assess annual trends in incidence, cleft type distribution, sex ratio, and associated anomaly rates. We also consider the influence of sociocultural changes, particularly delayed childbearing, on CL/P occurrence. 

This study aims to (1) describe long-term trends in the annual incidence of CL/P across the Tokai region (Aichi, Gifu, and Mie prefectures) over a 43-year period (1981-2023), (2) examine whether there were significant temporal changes in the overall CL/P incidence during this period, and (3) compare the regional incidence patterns with recent national and global epidemiological findings.

## Materials and methods

This retrospective epidemiological study utilized data from a CL/P surveillance system conducted by the Cleft Lip and Palate Center at Aichi Gakuin University Dental Hospital since 1981. The survey targeted all delivery-capable healthcare facilities (hospitals, clinics, and maternity centers) located in Aichi, Gifu, and Mie prefectures. These three prefectures constitute the Tokai region, situated in Central Honshu, Japan’s main island, between the Kanto region to the east (Tokyo area) and the Kansai region to the west (Osaka, Kyoto). According to the 2020 National Census, the Tokai region has a total population of approximately 10.1 million (Aichi, 7.5 million; Gifu, 2.0 million; and Mie, 1.5 million), accounting for about 8% of Japan’s total population. Each annual survey period ran from January 1 to December 31, excluding stillbirths and terminations, with data collected per calendar year.

A mailed questionnaire was distributed annually to all eligible obstetric providers. The list of institutions was updated each year based on the Ministry of Health, Labour and Welfare’s designated maternal protection facility registry and local government facility listings. Respondents were asked to report the following items based on their routine clinical diagnostic practice, as no additional training or standardized diagnostic definitions were provided: (1) total live births in the year, (2) presence and type of any congenital anomalies (based on routine clinical diagnosis at each facility; no specific international coding system, such as ICD-11, was mandated), (3) occurrence of CL/P, (4) cleft subtype (isolated lip, isolated palate, or combined lip and palate), (5) sex of the affected infant, and (6) presence of associated anomalies (e.g., cardiac malformations, limb defects, chromosomal abnormalities).

The questionnaires were paper-based and, once completed, were transcribed into a dedicated electronic database. To ensure data quality, all entries were checked by a second member of the research team, and any discrepancies were resolved by re-checking the original questionnaire forms. Annual incidence rates of CL/P (per 10,000 live births), type-specific distributions, sex ratios, geographic breakdowns, and rates of associated anomalies were calculated. Statistical analyses were performed using IBM SPSS Statistics for Windows, Version 26.0 (Released 2018; IBM Corp., Armonk, NY, US). Differences in categorical variables such as sex ratios and trends were assessed with chi-square tests or Fisher’s exact tests when appropriate. A p-value of less than 0.05 was considered statistically significant.

To evaluate the potential impact of non-response bias, we performed a sensitivity analysis assuming different incidence rates in non-responding institutions.

Institutional review and ethical approval were obtained from the Japan Oral Care Association Ethics Committee.

## Results

Survey response rate

**Figure 1:**
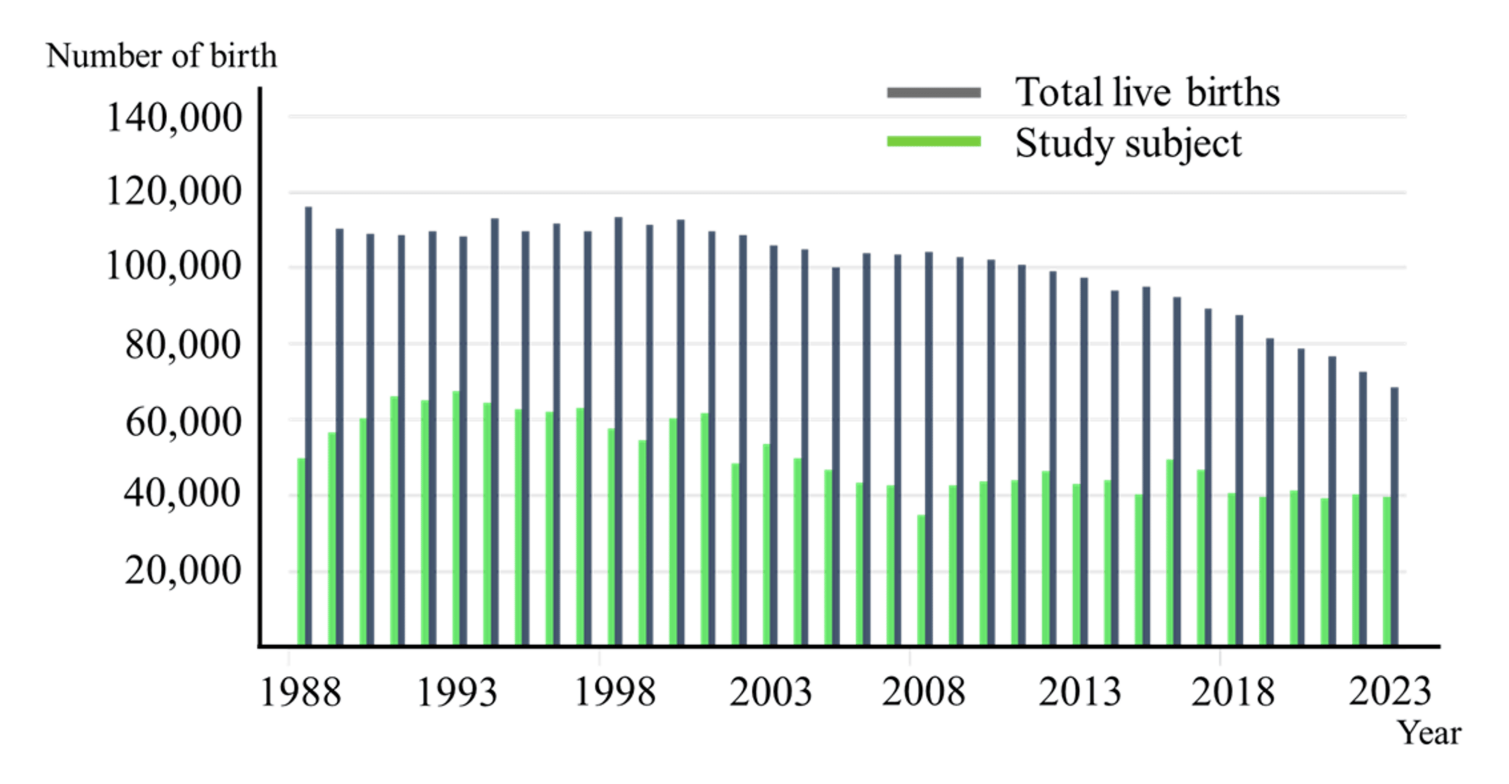
Trends in the number of births and study registrants in the three Tokai prefectures Birth numbers have been declining annually, whereas the questionnaire response rate has shown an upward trend, reaching around 60% in recent years. "Total birth" refers to the number of live births in the three Tokai prefectures as reported by the Ministry of Health, Labour and Welfare. "Study subject" refers to the number of live births at the institutions that responded to the survey.

The survey response rate was defined as the proportion of newborns born at facilities that returned the annual survey form, out of all newborns born at facilities capable of providing delivery care identified in the public registry in Aichi, Gifu, and Mie Prefectures. The rate was approximately 30% in 1981, exceeded 40% after 1988, and stabilized between 50% and 60% from the 2000s onward (Figure 1). When non-responding institutions were assumed to have the same incidence as responding ones, the overall incidence remained 0.15%. If non-responding institutions were assumed to have 20% lower or 20% higher incidence rates, the overall incidence changed only marginally, ranging from 0.14% to 0.16%. These results indicate that the potential impact of non-response bias on our overall incidence estimates is minimal.

Trends in CL/P incidence

**Figure 2:**
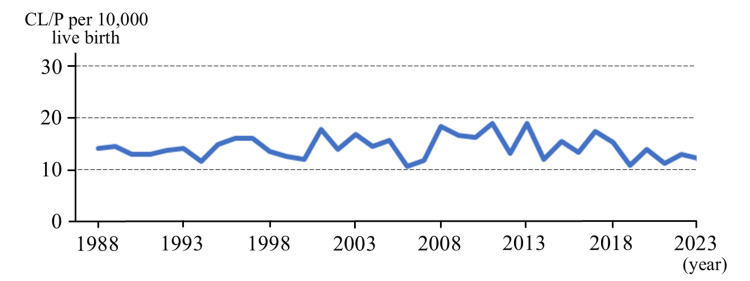
Annual incidence rates of CL/P per 10,000 live births in Aichi, Gifu, and Mie Prefectures, Tokai region, Japan (1988-2023) Data from 1981-1987 are not plotted because some facilities had incomplete reporting in those early years; reporting completeness improved after 1988 (survey response rate exceeded ~40%); therefore, the figure presents annual rates from 1988 onward for clearer trend interpretation. The incidence rate of CL/P is 10-20 per birth, and no significant change has been observed over the past 40 years.

From 1981 to 2023, annual live birth counts in Aichi initially exceeded 70,000 but declined to under 50,000 by 2023. This reduction reflects the nationwide demographic trend of declining fertility in Japan, largely due to delayed marriage [9], reduced family size, and a shrinking population of women of childbearing age, rather than region-specific factors or selective termination (Table 1). CL/P incidence remained between 10 and 20 per 10,000 births (1 in 500-1,000 births). Gifu and Mie experienced similar birth declines, to around 10,000 by 2023, with CL/P incidence across prefectures ranging between 8 and 14 per 10,000 births in Gifu and 4 and 23 per 10,000 births in Mie (Table 2). The overall incidence across the three prefectures was 14.45 per 10,000 births (95% CI: 13.89-15.00). No statistically significant upward or downward trends were observed within each prefecture or region-wide (chi-square test: Aichi, p = 0.40; Gifu, p = 0.10; Mie, p = 0.89; overall region: p = 0.89) (Figure 2).

**Table 1:** Trends in the average age at marriage and childbirth in Japan over the past 40 years

Year	Average age at first marriage for women	Average age at first birth	Average age at second birth	Average age at third birth
1975	24.7	25.7	28.9	30.3
1980	25.2	26.4	28.7	30.6
1985	25.5	26.7	29.1	31.4
1990	25.9	27.0	29.5	31.8
1995	26.3	27.5	29.8	32.0
2000	27.0	28.0	30.4	32.3
2005	28.0	29.1	31.0	32.6
2010	28.8	30.5	31.8	33.2
2015	29.4	31.1	32.5	33.5
2019	29.6	31.2	32.7	33.8

**Table 2:** Study subjects and CL/P patients in the three prefectures between 1988 and 2023 CL/P: cleft lip and/or palate.

Year	Aichi Prefecture		Gifu Prefecture		Mie Prefecture	
	Study subject	Number of CL/P cases	Study subject	Number of CL/P cases	Study subject	Number of CL/P cases
1988	33,545	40	21,791	18	18,931	13
1989	40,091	58	20,614	12	18,183	13
1990	34,034	44	20,295	18	17,918	17
1991	39,078	45	20,033	25	17,519	16
1992	44,094	54	20,347	23	17,686	13
1993	41,569	71	20,017	15	17,368	10
1994	41,462	50	20,623	10	18,144	15
1995	38,577	58	20,187	20	17,500	16
1996	37,100	57	20,546	26	17,780	17
1997	39,912	62	19,930	25	17,660	14
1998	33,351	46	20,447	18	17,829	14
1999	33,271	56	20,151	9	17,375	4
2000	38,707	53	20,276	6	17,726	14
2001	37,632	62	19,603	25	17,094	23
2002	29,449	48	19,617	11	17,190	9
2003	33,112	58	19,156	19	16,497	13
2004	29,537	48	18,363	12	16,287	13
2005	27,243	41	17,706	16	15,345	16
2006	26,121	27	18,092	13	15,816	6
2007	24,441	34	17,696	11	15,716	6
2008	22,435	38	17,506	16	15,633	10
2009	28,399	46	17,327	9	15,614	16
2010	27,846	44	16,887	13	15,262	14
2011	31,069	52	16,851	21	15,080	10
2012	34,281	41	16,496	11	14,729	9
2013	33,041	67	16,000	7	14,514	8
2014	31,598	37	15,138	7	13,727	9
2015	29,483	47	15,464	11	13,950	4
2016	34,013	43	14,831	13	13,202	10
2017	31,361	63	14,039	12	12,663	7
2018	29,039	52	13,719	7	12,582	3
2019	26,366	28	12,776	9	11,690	6
2020	29,118	45	12,092	6	11,141	5
2021	26,540	30	11,730	9	10,980	5
2022	28,524	43	11,124	4	10,489	5
2023	27,524	39	10,469	1	9524	8

Cleft type and sex ratio

**Figure 3:**
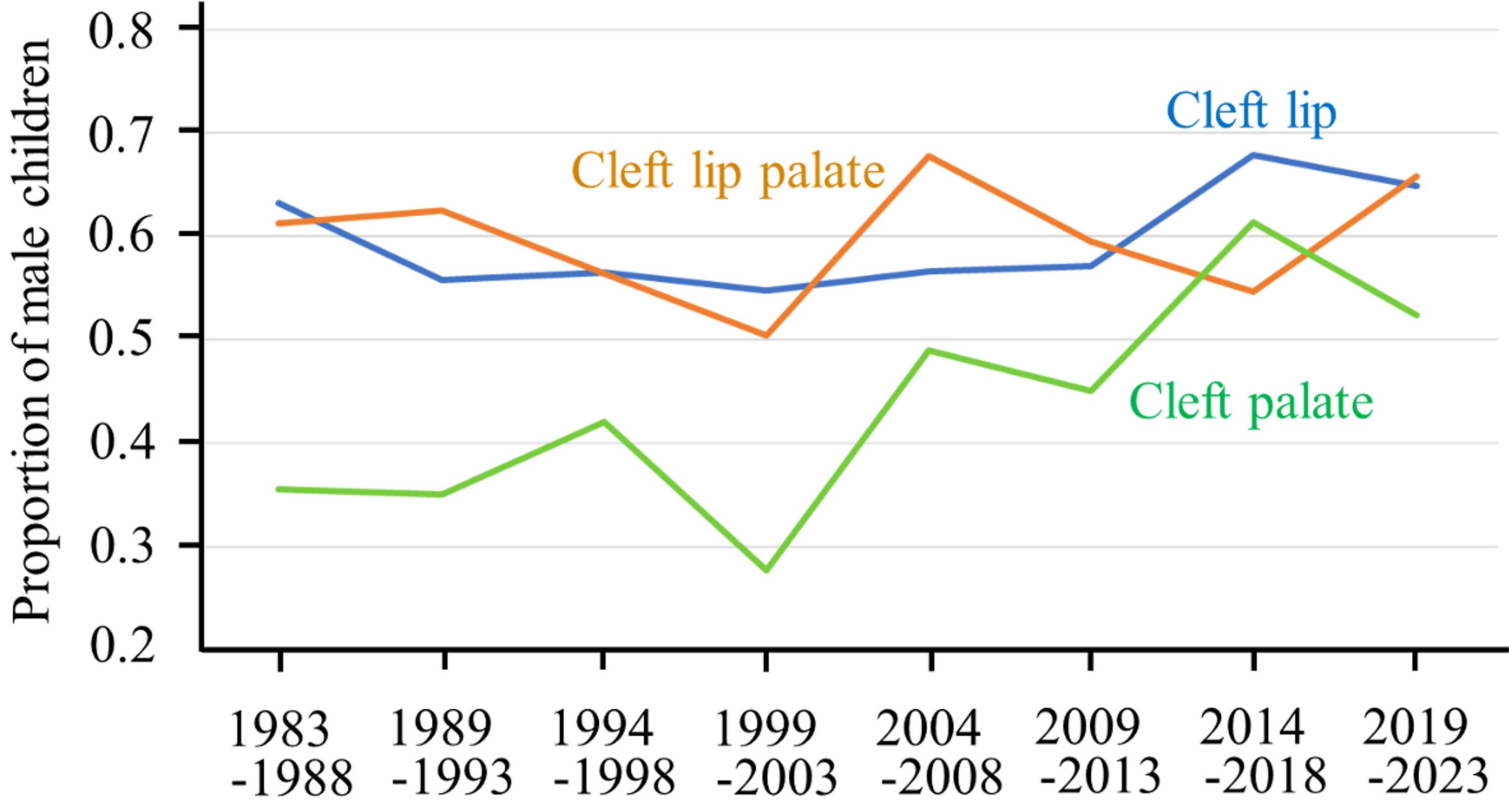
Sex differences among children with CL, CLP, and CP CL and CLP occur more frequently in males, while CP is more common in females. However, in recent years, the gender difference in CP has become less distinct. CL: cleft lip, CLP: cleft lip with cleft palate, CP: cleft palate.

Subtype distributions were consistent over time. Males predominated in cases of CL and CLP, while historical data showed a female predominance in CP (Figure 3).

Associated congenital anomalies

**Figure 4:**
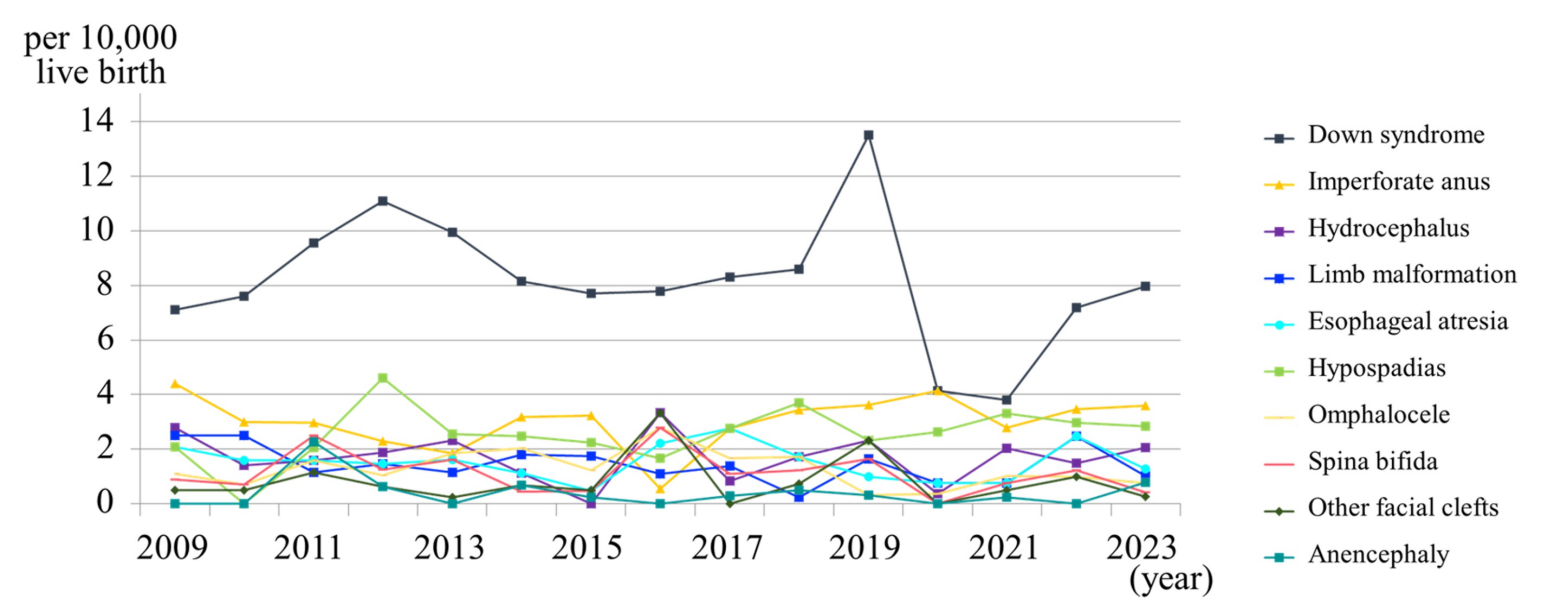
Annual trends in the incidence rates of other major congenital anomalies in the Tokai region, Japan (2009-2023) Although the incidence of Down syndrome shows considerable annual variation, the overall incidence rates of congenital anomalies have not changed during the last 15 years.

From 2009 onward, additional data were collected on cardiac, limb, neural tube, and chromosomal anomalies. Between 2009 and 2023, their prevalence remained stable, with no significant year-to-year changes or upward trends in any category (chi-square test for trend, p > 0.05) (Figure 4).

## Discussion

As reported in the Results section, the annual survey response rate gradually increased from approximately 30% in 1981 to stabilize between 50% and 60% from the 2000s onward. The gradual improvement in response rates may have been facilitated by enhanced engagement efforts, including distribution of annual summary booklets, collaboration with the Japanese Cleft Palate Foundation (JCPF), provision of feeding support materials, and performance-based feedback and recognition incentives. This likely contributed to the completeness and reliability of the collected data over time.

From 1981 to 2023, annual live birth counts in Aichi declined gradually. The decline in annual live births observed in the Tokai region mirrors Japan’s nationwide demographic shift, driven mainly by lower fertility rates and changes in reproductive behavior, such as delayed marriage and childbearing. In this longitudinal regional surveillance study spanning over four decades, the incidence of CL/P in the Tokai region remained remarkably stable (0.10%-0.20%), despite demographic changes such as declining birth rates, delayed marriage, and rising maternal age [9] (Table 1). This stability suggests that CL/P is primarily influenced by genetic predisposition and less by short-term environmental or social shifts. Moreover, despite improvements in prenatal diagnostic capabilities, termination based solely on CL/P is not common in Japan, potentially accounting for the unchanging birth incidence. Although demographic and social factors such as delayed marriage and rising maternal age have changed markedly over the study period, the present data do not allow direct causal inferences regarding their relationship with CL/P incidence. These observations should be interpreted as concurrent trends rather than causal associations.

The sex distribution patterns observed in the Tokai region, male predominance in CL/CLP and female predominance in CP, are consistent with those reported across other regions of Japan and in international studies. Although the reversal of the CP sex ratio after 2004 was observed, the sample size for CP cases was relatively small. This represents a notable observation that warrants further investigation, as ongoing detailed analyses may provide additional insights.

Although minor fluctuations in the CP sex ratio were observed, the number of cases was small, and detailed subtype analyses are being conducted in a separate study. In this report, we focus on overall CL/P incidence trends. The stable incidence over 43 years provides important information for healthcare planning, perinatal counseling, and policy-making, as it allows for predictable allocation of resources and sustained monitoring efforts.

Furthermore, our expanded surveillance from 2009 onward indicates that the rates of other major congenital anomalies (e.g., cardiac, limb, chromosomal) have remained constant, which may reflect sustained standards of perinatal and prenatal care in the region. The stable prevalence of other major congenital anomalies from 2009 to 2023 may reflect the consistent quality of perinatal care and prenatal diagnostic practices in the Tokai region. Despite demographic shifts, such as increased maternal age, advances in maternal health management and stable reporting systems likely contributed to the absence of significant temporal changes.

This study has several limitations. First, although the questionnaire response rate has improved, the data still only cover approximately 60% of all births. The study does not include all births in the Tokai region, as only deliveries at responding institutions were captured. Although sensitivity analyses indicate minimal impact on overall incidence estimates, the potential for non-response bias cannot be completely excluded. Additionally, because the cleft types were not diagnosed through direct clinical examination of the patients and were reported based on routine clinical diagnostic practice without standardized definitions (e.g., ICD-11), there may be some uncertainty regarding diagnostic accuracy. In particular, it is possible that minor forms such as submucous CP or soft palate clefts were overlooked, which could have led to a slight underestimation of the true incidence rates. Thus, some degree of misclassification bias cannot be excluded, which represents an inherent limitation of relying on provider-reported questionnaire data. Furthermore, our study is limited by the absence of detailed maternal, socioeconomic, and environmental data, which precludes assessment of potential genetic-environmental interactions influencing CL/P risk.

Nevertheless, the strengths of this study lie in its extensive geographic coverage, including all delivery facilities across the three prefectures of the Tokai region, and its consistent methodology maintained over more than 40 years. As such, it provides one of the most comprehensive and reliable regional CL/P datasets in Japan. We intend to continue this surveillance in the future.

## Conclusions

Using data from a sustained 43-year regional surveillance program in Tokai, Japan, this study demonstrates that CL/P incidence has remained stable at approximately 10-20 per 10,000 live births, even amid societal shifts such as rising maternal age and lower birth rates. The observed reversal in CP sex ratio post‑2004 requires additional investigation. This long-term surveillance demonstrates stable CL/P incidence despite demographic shifts, such as declining birth rates and rising maternal age. While these changes occurred concurrently, causal relationships cannot be inferred from the present data. This research underscores the importance of long-term, regionally oriented congenital anomaly monitoring to inform public health planning, genetic counseling strategies, and perinatal care improvements. Ongoing data collection and analysis, ideally including comparisons across other regions and internationally, are strongly encouraged.
